# About two unusual cases of pancreatic primary squamous cell carcinoma

**DOI:** 10.1093/jscr/rjae420

**Published:** 2024-07-26

**Authors:** Ismail Boujida, Kenza Horache, Sabrine Derqaoui, Ahmed Jahid, Fouad Zouaidia, Omar El-Aoufir, Zakia Bernoussi, Kaoutar Znati

**Affiliations:** Department of Pathology, Ibn Sina University Hospital Center, 10100, Rabat, Morocco; Faculty of Medicine and Pharmacy, Mohammed 5 University, 10100, Rabat, Morocco; Faculty of Medicine and Pharmacy, Mohammed 5 University, 10100, Rabat, Morocco; Department of Radiology, Ibn Sina University Hospital Center, 10100, Rabat, Morocco; Department of Pathology, Ibn Sina University Hospital Center, 10100, Rabat, Morocco; Faculty of Medicine and Pharmacy, Mohammed 5 University, 10100, Rabat, Morocco; Department of Pathology, Ibn Sina University Hospital Center, 10100, Rabat, Morocco; Faculty of Medicine and Pharmacy, Mohammed 5 University, 10100, Rabat, Morocco; Department of Pathology, Ibn Sina University Hospital Center, 10100, Rabat, Morocco; Faculty of Medicine and Pharmacy, Mohammed 5 University, 10100, Rabat, Morocco; Faculty of Medicine and Pharmacy, Mohammed 5 University, 10100, Rabat, Morocco; Department of Radiology, Ibn Sina University Hospital Center, 10100, Rabat, Morocco; Department of Pathology, Ibn Sina University Hospital Center, 10100, Rabat, Morocco; Faculty of Medicine and Pharmacy, Mohammed 5 University, 10100, Rabat, Morocco; Department of Pathology, Ibn Sina University Hospital Center, 10100, Rabat, Morocco; Faculty of Medicine and Pharmacy, Mohammed 5 University, 10100, Rabat, Morocco

**Keywords:** squamous cell carcinoma, pancreatic neoplasm, immunohistochemistry

## Abstract

Pancreatic primary squamous cell carcinoma (PPSCC) is very uncommon. The major diagnostic method is histology, and it requires the exclusion of a metastasis from a different primary location (lung, esophagus…). Herein, we describe two cases of a PPSCC (one in the head and the other one in the tail and the body of the pancreas) with a brief review of literature. When it comes to the poorly differentiated PPSCC, immunohistochemistry (IHC) is crucial. Regretfully, there is currently no unanimity on treatment, and the outcome is dismal.

## Introduction

Pancreatic primary squamous cell carcinoma (PPSCC) is an extremely rare malignant tumor of the exocrine pancreas, accounting for between 0.5% and 5% of all pancreatic cancers [1, 2]. Its diagnosis is confirmed by the absence of a glandular component and after exclusion of a primary squamous cell carcinoma (SCC) from another location.

We present here two cases of PPSCC in two North African men, in a hospital in Rabat, in order to illustrate the epidemiological, clinical, and histological features of this unusual entity.

## Cases

**Figure 1:**
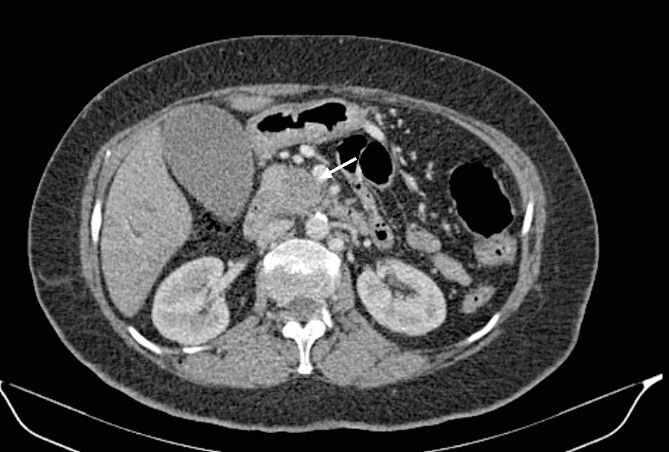
Low-enhancing poorly defined mass in the pancreatic head with surrounding fat stranding (arrow).

**Figure 2:**
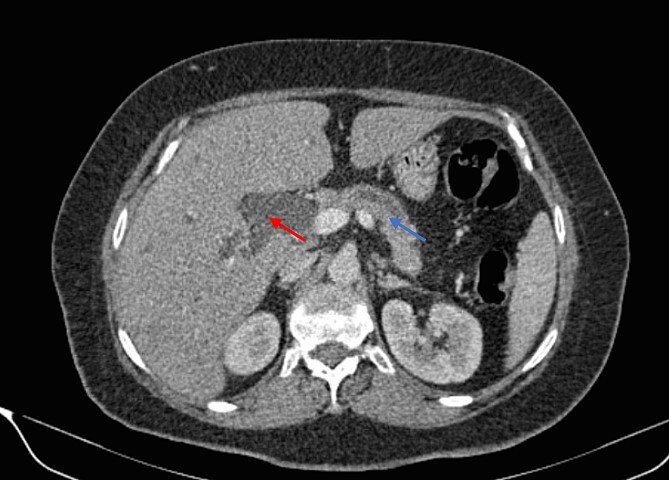
Parenchymal atrophic changes and main pancreatic duct dilatation distal to the mass (blue arrow). Extra and intrahepatic bile ducts are dilated (red arrow).

**Figure 3:**
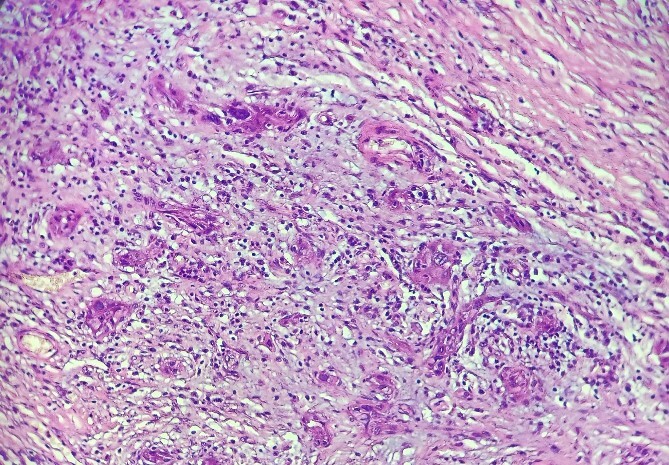
HE section: The tumor cells are so poorly differentiated that it is difficult to tell what the cell of origin is. The cells are discohesive with bizarre looking nuclei (×400).

**Figure 4:**
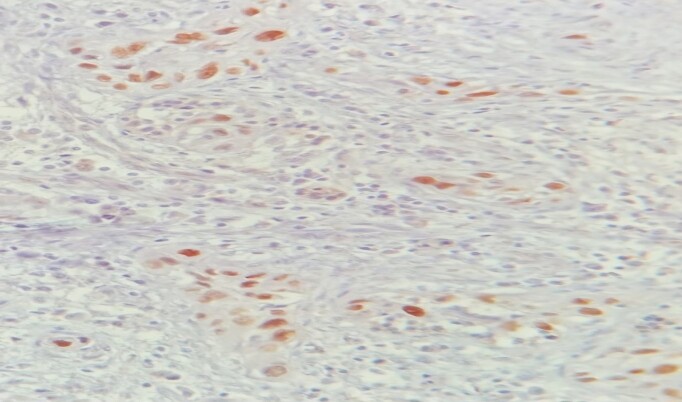
IHC: Diffuse nuclear staining of p40 by tumor cells (×400).

Mr. A. is a 65-year-old hypertensive patient presenting with acute epigastric pain associated with abdominal bloating. Biological settings showed amylasemia at 450 IU/L and lipasemia at 525 IU/L. Abdominal CT scan revealed heterogenous poorly defined mass in the pancreatic head with low enhancement (Figs 1 and 2). The CA 19–9 assay was 280 IU/L. Cephalic duodenopancreatectomy was performed. Histological examination of the operative specimen revealed a solid, trabecular, undifferentiated carcinomatous process (Fig. 3) expressing markers (CK(AE1-AE3) and p40) (Fig. 4), confirming the diagnosis of primary squamous cell carcinoma of the pancreas. The extension study showed no secondary localization.

**Figure 5:**
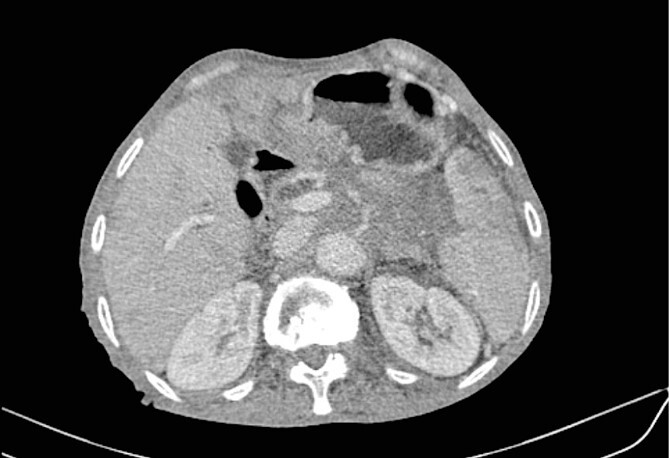
Low enhancing mass at pancreatic body and tail with internal necrotic changes. The mass encases the AMS and its branches and encases splenic vessels. Loss of the fat plane between the mass and adjacent spleen.

**Figure 6:**
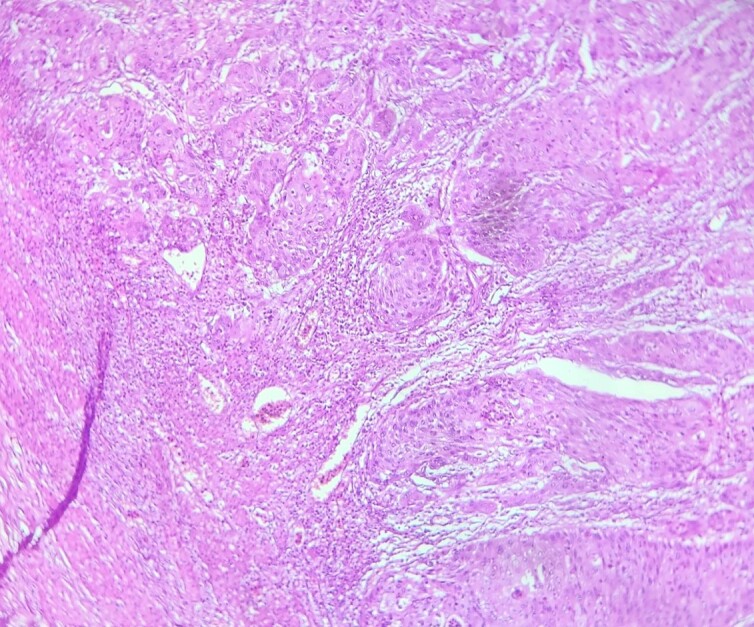
HE section showing nests and sheets of polygonal cells surrounded by fibrotic desmoplastic stroma (x100).

**Figure 7:**
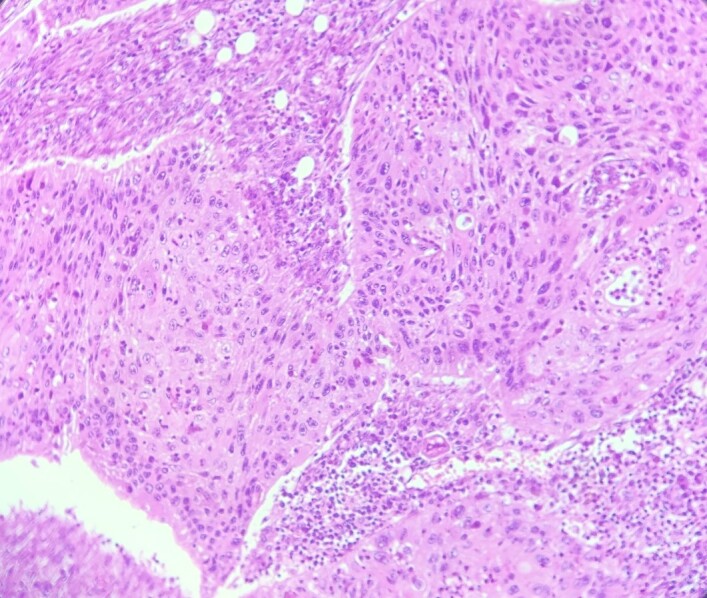
HE section: Neoplastic cells have densely chromatic enlarged nuclei with prominent nucleoli. They are enlarged with abundant eosinophilic cytoplasm and focal keratinization. Intercellular bridges are slightly visible (x200).

Mr. B. is a 59-year-old patient with no notable pathological history, presenting with melenas associated with marked weight loss. A fibroscopy revealed a thickened and ulcerated gastric mucosa. Abdominal and pelvic CT scans revealed a tissue process in the body and tail of the pancreas invading the splenic hilum, adjacent vessels ans gastric wall, measuring 11 cm long (Fig. 5). A caudal spleno-pancreatectomy enlarged to the colon, omentum and stomach was performed. Macroscopically, the tumor had a grayish–white appearance, poorly limited and indurated on palpation. Histologically, it was a well-differentiated, keratinizing squamous cell carcinoma of the tail of the pancreas (Figs 6 and 7), infiltrating the splenic hilum, gastric wall and colon.

## Discussion

PPSCC is a rare and fatal disease. Epidemiological studies report that it mainly affects elderly men [3].

A number of theories have been put forth to explain how PPSCC developed. The pancreas is normally completely free of squamous cells. Squamous metaplasia of the ductal columnar cells is frequently observed in inflammatory states, such as pancreatitis. In fact, it has been hypothesized that ductules that have experienced squamous metaplasia as a result of persistent inflammation are the source of pancreatic squamous cell carcinoma [4].

It could also be a mixed adenosquamous carcinoma in which the glandular components have vanished.

The tumor could as well develop from an already-existing adenocarcinoma with squamous metaplasia, or it may drift from a biopotential primitive able to evolve into either glandular adenocarcinoma or SCC [5].

Clinically, the most frequent manifestations are abdominal and back pain, weight loss, jaundice and vomiting [6], although PPSCC is usually asymptomatic and only diagnosed at advanced stages of the disease (Stages III and IV). However, in patients presenting with pancreatic tumors with elevated CEA or CA 19–9 levels, malignancy is strongly suspected and PPSCC cannot be ruled out.

Tumor enhancement on contrast CT, tumor blush patterns on angiography, endoscopic retrograde cholangiopancreatography, and positron emission tomography–computed tomography scan can be useful in establishing a diagnosis of PPSCC [7]. It has been reported that pancreatic squamous cell carcinoma and classical ductal adenocarcinoma can be distinguished from one another using the enhancement of the tumor on contrast-enhanced CT scans and the blush patterns of the tumor on angiography. These two radiographic abnormalities are most likely caused by squamous cell carcinoma’s hypervascularity [4].

It is diagnosed histologically, in the presence of well-differentiated or poorly differentiated squamous carcinomatous proliferation: The formation of whorls or ‘pearls’ with intercellular bridges, irregularly shaped nests and cords of epithelial cells, with eosinophilic cytoplasm. These histological findings are characteristic of squamous cell carcinoma [4]. The confirmation thus, is made by immunohistochemistry (p40, cytokeratin, p63); and by the absence of an associated glandular component [8]. Looking for a primitive tumor elsewhere is also crucial because there are no criteria for cytologic differentiation between primary and metastatic pancreatic squamous cell carcinoma [4].

Unfortunately, most patients are diagnosed at a later stage (75% Stage III and IV). For the latter, the evolution is fatal, with a median survival of 3 to 4 months.

It has been observed that the overall survival time for patients who refuse therapy is as little as three months. On the optimal therapeutic approaches, however, there was disagreement. Since pancreatic cancer has a 7% chance of survival, treating it can be extremely difficult. Pancreatic malignancies at stages I, IIA, and IIB are thought to be treatable with surgery. Surgical resection is the only treatment that is thought to be potentially curative. Surgery is frequently used in conjunction with adjuvant radiotherapy and chemotherapy [7].

In contrast to pancreatic adenocarcinoma, there are only a few case series in the literature, so no effective therapeutic consensus exists to date [8].

## Conclusion

To sum up, PPSCC is an uncommon tumor with a really poor outlook. Before declaring it primary, it is always important to take into account and thoroughly rule out a spread from other primary squamous cell carcinomas.

Although there is currently no known treatment for PPSCC, surgery is still the gold standard when the tumor is still in its early stages.
